# Bevacizumab promotes active biological behaviors of human umbilical vein endothelial cells by activating TGFβ1 pathways **via** off-VEGF signaling

**DOI:** 10.20892/j.issn.2095-3941.2019.0215

**Published:** 2020-05-15

**Authors:** Xiaoling Zhang, Yan Zhang, Yanan Jia, Tingting Qin, Cuicui Zhang, Yueya Li, Chengmou Huang, Zhujun Liu, Jing Wang, Kai Li

**Affiliations:** ^1^Department of Thoracic Oncology, Tianjin Medical University Cancer Institute and Hospital, National Clinical Research Center for Cancer, Key Laboratory of Cancer Prevention and Therapy, Tianjin, Tianjin’s Clinical Research Center for Cancer, Tianjin 300060, China; ^2^Department of Radiotherapy, Lanzhou University Second Hospital, Lanzhou 100040, China; ^3^Department of Oncology, The First Affiliated Hospital of Hainan Medical University, Haikou 570102, China

**Keywords:** HUVEC, CD105, bevacizumab, anlotinib, TGFβ

## Abstract

**Objective:** Bevacizumab is a recombinant humanized monoclonal antibody that blocks vascular endothelial growth factor (VEGF) with clear clinical benefits. However, overall survival of some cancer types remains low owing to resistance to bevacizumab therapy. While resistance is commonly ascribed to tumor cell invasion induced by hypoxia-inducible factor (HIF), less attention has been paid to the potential involvement of endothelial cells (ECs) in vasculature activated by anti-angiogenic drugs.

**Methods:** Human umbilical vein ECs (HUVECs), bEnd.3 cells, and mouse retinal microvascular ECs (MRMECs) were treated with bevacizumab under conditions of hypoxia and effects on biological behaviors, such as migration and tube formation, examined. Regulatory effects on TGFβ1 and CD105 (endoglin) were established *via* determination of protein and mRNA levels. We further investigated whether the effects of bevacizumab could be reversed using the receptor tyrosine kinase inhibitor anlotinib.

**Results:** Bevacizumab upregulated TGFβ1 as well as CD105, a component of the TGFβ receptor complex and an angiogenesis promoter. Elevated CD105 induced activation of Smad1/5, the inflammatory pathway and endothelial–mesenchymal transition. The migration ability of HUVECs was enhanced by bevacizumab under hypoxia. Upregulation of CD105 was abrogated by anlotinib, which targets multiple receptor tyrosine kinases including VEGFR2/3, FGFR1-4, PDGFRα/β, C-Kit, and RET.

**Conclusions:** Bevacizumab promotes migration and tube formation of HUVECs *via* activation of the TGFβ1 pathway and upregulation of CD105 expression. Anlotinib reverses the effects of bevacizumab by inhibiting the above signals.

## Introduction

Bevacizumab is a recombinant humanized monoclonal antibody targeting VEGF-A that has beneficial clinical effects^1,2^. However, improvements in progression-free survival (PFS) but not overall survival (OS) have been reported in a number of clinical trials^3–5^, for instance, in patients with progressive glioblastoma^6^. In another study, bevacizumab failed to reduce tumor growth, instead exacerbating brain tumor invasion in mice bearing glioma^7^, indicating a capacity to stimulate malignant behavior of tumor cells. Glioblastoma and colorectal cancer recurrence, characterized by highly infiltrative behavior, have additionally been documented after bevacizumab treatment^8–10^. These results highlight the urgent need to identify “high-risk individuals” prone to progressive disease induced by unregulated anti-angiogenic treatment to avoid unproductive therapy.

Besides malignant cells, another non-negligible factor in resistance to anti-angiogenesis therapy is potentially the endothelial cells (ECs) of micrangium around the tumors. However, it remains to be established whether endothelial cells of vessels undergo a similar phenomenon as neoplastic cells. The potential malignant behavior of ECs has rarely been explored as they are considered mature, gene-stable cells that lack features of malignancy. However, in response to pro-angiogenic signals, ECs become active and motile with protruding filopodia and retain high plasticity, responding to angiogenic tumor expansion^11^. Additionally, tumors can “hijack” pre-existing blood vessels into the vasculature whereby ECs acquire “motivated status” with endothelial–mesenchymal transition (Endo-MT) associated with TGFβ-CD105-Smad and Notch signaling to boost neointima formation and leukocyte transmigration^12–15^. In the current study, treatment of human umbilical vein EC (HUVEC) cells with bevacizumab under hypoxia led to increased migration and tube formation, similar to activated ECs with EMT phenotype in previous reports^16,17^. Therefore, the issue of whether normal vessel ECs can be activated by aberrant stimulation and the underlying mechanisms need further exploration.

TGFβ1 in ECs activates Sma- and Mad-related (Smad) proteins. Smad and JNK signaling in the TGFβ1 pathway promote Endo-MT^13,15^ and inflammation in rat peritoneal mesothelial cells^18^. The extracellular and cytoplasmic domains of the auxiliary TGFβ receptor CD105 (endoglin) interact with alk1 in ECs. Alk1 and alk5 (different TGFβ superfamily receptor I types) in ECs are proposed to regulate the balance between proliferation and quiescence^19^, whereby binding of CD105 with alk1-Smad1/5/8 activates ECs in association with Endo-MT^13^. Additionally, elevated CD105 is associated with inflammatory infiltration *in vivo*^20^ and endogenous secreted CCL20 levels are increased in oral cancer cells due to CD105 stimulation^21^. Here, we further focused on elucidating the mechanisms underlying the impact of bevacizumab on downstream inflammatory factors of the TGFβ-CD105 pathway in HUVECs.

## Materials and methods

### Cell culture and reagents

HUVECs (Peking Union Medical College Cell Bank, Beijing, China), bEnd.3 (Nankai University, Tianjin, China) and MRMECs (Tianjin Medical University Eye Hospital, Tianjin, China) were cultured in DMEM containing 10% FBS. Hypoxia (< 1% O_2_) was induced with a modular incubator chamber (Billups-Rothenberg, San Diego, CA, USA). Bevacizumab was purchased from Roche (H0160) and anlotinib was a gift from Nanjing Chia Tai Tianqing Company (Nanjing, China).

### *In vitro* angiogenesis assay

HUVECs were treated with various concentrations of bevacizumab for 24 h under hypoxia conditions. Next, cells were seeded in a 48-well plate pre-coated with 150 μL matrigel (BD Biosciences, Bradford, MA, USA) at a density of 4 × 10^4^ cells/well. After 5 h, images of enclosed tubes were obtained with an inverted phase-contrast microscope (Leica DMI6000B, 50× magnification).

### *In vivo* angiogenesis assay

Female BALB/c-nu mice 6–8 weeks old were purchased from the Model Animal Center of Nanjing University (Nanjing, China). In keeping with a previous protocol^22^, HUVECs (2 × 10^7^ cells/mL) were resuspended on ice in phenol red-free matrigel solution, mixed with different doses of bevacizumab (0, 10, and 100 μg/mL) together with 1 μg/mL VEGF (PeproTech), and implanted subcutaneously into BALB/c-nu mice. Mice were divided into three groups intraperitoneally injected with 0, 5, and 50 mg/kg bevacizumab twice a week for 1 month. Images of the matrigel were obtained and fixed with 4% paraformaldehyde for CD105 immunohistochemistry (ab137389, anti-human CD105 antibody does not cross-react with mouse CD105). Experiments were replicated using 4 mice per group. To confirm the efficacy of bevacizumab on endothelial cells, experiments on bEnd.3 cells were additionally performed.

### Migration assay

HUVECs (5 × 10^4^ cells/well) were seeded in transwell inserts (8 μm, Corning Inc, NY, USA) with DMEM containing 20% FBS for 8 h. Cells were pretreated with 0–160 μg/mL bevacizumab under hypoxia or normal oxygen conditions for 24 h. Cells were stained with Crystal violet (Beyotime, Haimen, Jiangsu, China) and digital images (100× magnification) of the insert undersides obtained under a microscope (ECLIPSE TS100, Nikon, Tokyo, Japan).

### RT-PCR and ELISA

#### RT-PCR

Total RNA was extracted with TRIzol (Qiagen, Valencia, CA, USA) and cDNA generated by reverse transcription using a first-strand cDNA synthesis kit (TransGen Biotech, Beijing, China), RT-PCR was performed using the TransScript^®^ RT/RI Enzyme Mix, 2×TS ReactionMix. After that, quantitative real-time PCR was performed using the TransStart Top Green qPCR SuperMix (TransGen Biotech, Beijing, China). The reactions were performed under the following conditions as suggested by the manufacturer: 94 °C for 30 s, followed by 40 cycles of 94 °C for 5 s and 60 °C for 30 s, followed by a dissociation protocol. Single peaks in the melting curve analysis indicated specific amplicons. Results were expressed as relative fold change calculated using the delta CT method. The primers used in this study are listed in **Supplementary Table S1**.

#### ELISA

HUVECs were treated with 10 and 100 μg/mL bevacizumab (24 h), anlotinib 10 μM (24 h), bevacizumab (100 μg/mL for 8 h) and anlotinib (10 μM for 16 h) sequentially. The supernatant was collected to determine the TGFβ1 concentration. ELISA was conducted according the manufacturer’s instructions (Dakewe, Shenzhen, China).

### MTT assay

HUVECs were transfected with CD105 siRNA for 24 h, plated into 96-well plates (2,000 cells/well), and incubated overnight with bevacizumab (100 μg/L). Next, 20 μL/well MTT (Solarbio Beijing, 5 mg/mL dissolved in PBS pH 7.4) was added to the plates. After 4 h, 150 μL DMSO was added, followed by shaking for 20 min. The plate was read using a Microplate Reader (Bio-Rad Laboratories, Hercules, CA, USA) at a wavelength of 490 nm.

### Western blot

HUVECs were homogenized, subjected to 12% SDS/PAGE, and subsequently transferred to PVDF membrane (Millipore, USA). Following blockage with 5% non-fat milk for 1 h at room temperature (RT), membranes were incubated with primary antibody overnight at 4 °C. The next day, blots were incubated with secondary antibody (1:5000 dilution, Santa Cruz) for 1 h at RT and developed using an ECL chemiluminescence reagent kit (Millipore, MA, USA). Antibodies used were as followed: CD105, CCL20 (Abcam, USA); Smad5, smad1 (ABclonal, China); IL1B, beta-Actin (Cell Signaling Technology, USA); Goat anti-rabbit antibody IgG-HRP (SantaCruz, USA).

### RNA interference and immunofluorescence

#### RNA interference

CD105 siRNA was synthesized by Ribobio Company (Guangzhou, China). The siRNA primer sequences are listed in **Supplementary Table S1**. HUVECs were transfected with siRNA at a final concentration of 50 nmol/L using Lipofectamine 2000 (Invitrogen).

#### Immunofluorescence

HUVECs were treated with different concentrations of bevacizumab under conditions of hypoxia. Cells were fixed with 4% paraformaldehyde for 10 min at RT, blocked with 10% goat serum for 1 h, and incubated at 4 °C overnight with CD105 (1:200, Abcam, ab107595), followed by Alexa Fluor 546 (1:200, Invitrogen, A-11035) for 1.5 h at RT. Cell nuclei were stained with DAPI and images acquired with an Axiovert 200 microscope (Carl Zeiss, Thornwood, NY, USA).

### Statistical analysis

Results are expressed as means ± SD on the basis of triplicate experiments. ANOVA and Student’s *t*-test (two-tailed) were employed for statistical analysis of significant differences between groups. For immunochemistry score analysis, the non-parametric test was used. *P* values *<* 0.05 were considered statistically significant.

## Results

### Bevacizumab promotes HUVEC migration and tube formation ***in vivo*** and ***in vitro***

**Figure 1:**
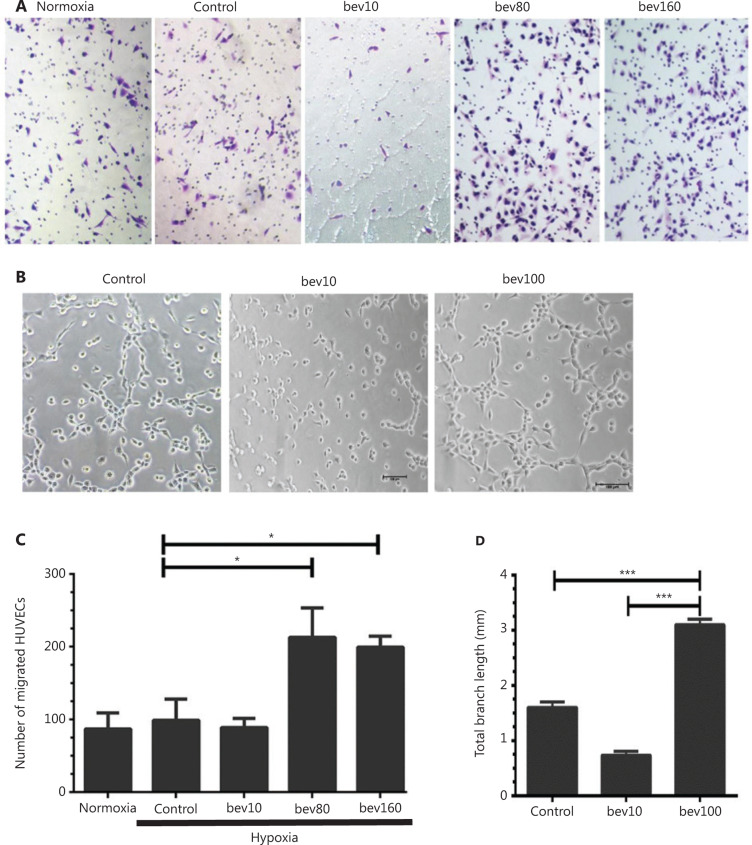
High concentration of bevacizumab (100 μg/mL) enhances migration and tube formation of HUVECs* in vitro*. (A) Typical images of migrating HUVECs treated with bevacizumab under hypoxia conditions (hypoxia: O_2_ < 1%, 5% CO_2_, 94% N_2_; control: bevacizumab 0 μg/mL; bev10: bevacizumab 10 μg/mL, bev100: bevacizumab 100 μg/mL; normoxia: normal oxygen vehicle: 21% O_2_, 5% CO_2_, 74% N_2_); magnification, ×100. (B) Images of canal-like tubules formed by HUVECs treated with bevacizumab under hypoxia; magnification, ×50. (C) Quantitative analysis of migrating HUVECs treated with different doses of bevacizumab under hypoxia. Data represent mean ± SD of three independent experiments; *P < 0.05; one-way ANOVA. (D) Average total branching lengths of canal-like tubules formed by HUVECs treated with bevacizumab under hypoxia. Data represent mean ± SD, *P < 0.05; **P < 0.01; ***P < 0.001; one-way ANOVA.

HUVECs were treated with 0–160 μg/mL bevacizumab under hypoxia or normal oxygen conditions for 24 h. Following treatment with 80 or 160 μg/mL bevacizumab in normoxia conditions (**Supplementary Figure S1A** and **S1B**), migration of HUVECs was decreased relative to the control group. However, under hypoxia, both concentrations of bevacizumab promoted cell migration to a significant extent (**Figure 1A** and **1C**, *P* < 0.05). In view of the finding that migration of HUVECs was activated by both 80 and 160 μg/mL bevacizumab, we employed a fixed concentration of 100 μg/mL for subsequent experiments^23^.

**Figure 2:**
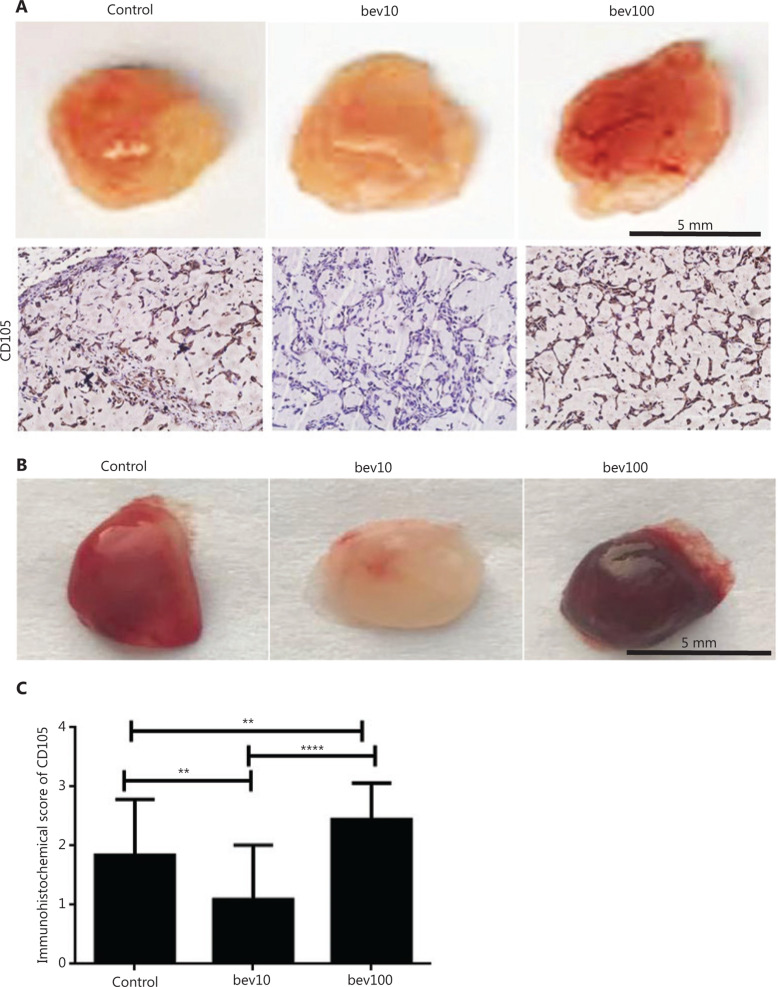
High concentration of bevacizumab (100 μg/mL) accelerates angiogenesis of HUVECs and bEnd.3 *in vivo*. (A) Comparison of blood vessel formation in matrigel (400 μL) plugs in female nude mice (*n* = 4 per group) by bev (bevacizumab: 0 μg/mL, 10 μg/mL and 100 μg/mL in matrigel). Mixed matrigel containing HUVECs, bevacizumab, and VEGFA was subcutaneously injected into mice. Mice were intraperitoneally injected with 0, 5, and 50 mg/kg bevacizumab twice a week for 1 month. The image shows matrigel separated from mice, with darker red indicative of higher blood content in vasculature in the gel. CD105 expression (brown: CD105^+^, the antibody was only reactive to human endothelial cells) determined *via* immunochemical assay. The CD105^+^ stain was stronger in HUVECs treated with high concentrations of bevacizumab than those treated with low concentrations of bevacizumab. (B) Comparison of blood vessel formation in matrigel (400 μL) plugs in female nude mice (*n* = 4 per group) from control (bevacizumab: 0 μg/mL in matrigel), bev10 (bevacizumab: 10 μg/mL in matrigel), and bev100 (bevacizumab: 100 μg/mL in matrigel) groups. Mixed matrigel containing bEnd.3 cells, bevacizumab, and VEGFA was subcutaneously injected into mice, followed by intraperitoneal injection with 0, 5, or 50 mg/kg bevacizumab twice a week for 9 days. The image shows matrigels separated from mice, with darker red indicative of higher blood content in vasculature in the gel. (C) Histogram displaying immunochemistry scores of CD105 in matrigel containing HUVECs (*n* = 22 per group, data represent mean ± SD, **P < 0.01, ****P < 0.0001; non-parametric test).

Tube length was significantly greater in the 100 μg/mL bevacizumab group than in the control group (**Figure 1B** and **1D**, *P* < 0.001). The tube formation experiment *in vivo* showed that 100 μg/mL bevacizumab promoted angiogenesis of HUVECs (**Figure 2A**) as well as bEnd.3 cells (**Figure 2B**). To determine the effects of bevacizumab on CD105 expression, immunohistochemical staining was conducted, which revealed upregulation of CD105 in the 100 μg/mL treatment group, compared to the control and 10 μg/mL treatment groups (**Figure 2A** and **2C**, *P* < 0.01).

### Bevacizumab enhances expression of CD105

**Figure 3:**
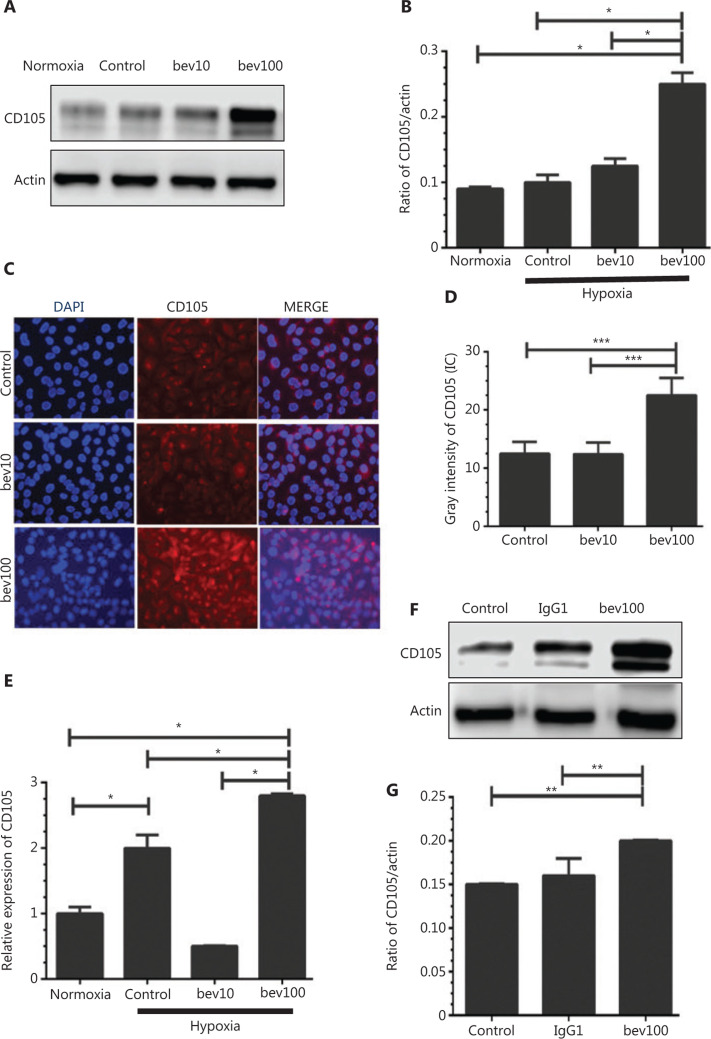
High concentration of bevacizumab (100 μg/mL) stimulates CD105 expression. (A) Western blot showing changes in CD105 protein levels following bevacizumab treatment under normoxia and hypoxia conditions (control: bevacizumab 0 μg/mL, bev10: bevacizumab 10 μg/mL, bev100: bevacizumab 100 μg/mL; normoxia: normal oxygen vehicle). (B) Quantitative analysis of CD105 protein levels following bevacizumab treatment under hypoxia. Data represent mean ± SD, *P < 0.05; one-way ANOVA. (C) Immunofluorescence of CD105 in HUVECs pre-stimulated with bevacizumab under hypoxia (control: bevacizumab 0 μg/mL, bev10: bevacizumab 10 μg/mL, bev100: bevacizumab 100 μg/mL), CD105 (red), and DAPI (blue). Magnification, ×200. (D) Quantitative analysis of fluorescence intensity of CD105^+^, ***P < 0.001; one-way ANOVA. (E) Changes in CD105 mRNA levels in response to bevacizumab under hypoxia conditions. Data represent mean ± SD, *P < 0.05; one-way ANOVA. (F) Western blot showing CD105 expression upon treatment with 100 μg/mL bevacizumab and isotype control IgG1 under hypoxia. (G) Quantitative analysis of CD105 protein expression following bevacizumab (100 μg/mL) and isotype control (IgG1) treatment. Data represent mean ± SD, **P < 0.01; one-way ANOVA.

**Figure 4:**
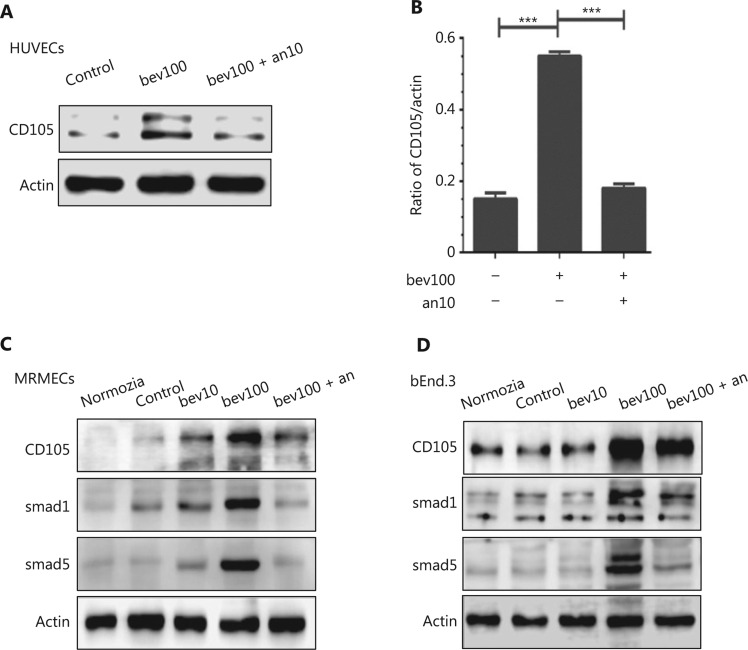
Bevacizumab upregulates CD105 and activates the TGFβ pathway in different cell lines under hypoxia, which is reversed by anlotinib regardless of the treatment sequence. (A) HUVECs were treated with bevacizumab (100 μg/mL, 24 h) or anlotinib (10 μM, 12 h) following pretreatment with bevacizumab (100 μg/mL, 12 h) under hypoxia. Anlotinib reversed bevacizumab-induced elevation of CD105. (B) Densitometric analysis of CD105 protein levels shown in (A). Data represent mean ± SD, ***P < 0.001, ANOVA. (C, D) MRMECs and bEnd.3 cells were treated with bevacizumab (0, 10, 100 μg/mL, 24 h) or anlotinib (10 μM, 12 h) following pretreatment with bevacizumab (100 μg/mL, 12 h) under hypoxia. Anlotinib reversed bevacizumab-induced elevation of CD105. Bevacizumab additionally enhanced Smad1 and Smad5 expression.

Both protein and mRNA levels of CD105 were significantly increased following treatment with 100 μg/mL bevacizumab for 24 h (**Figure 3A**, **3B**, and **3E**, *P* < 0.05). Results from immunofluorescence staining further validated bevacizumab-mediated upregulation of CD105 (**Figure 3C**). HUVECs stimulated with 100 μg/mL bevacizumab exhibited stronger fluorescent signals of CD105, compared to those treated with 10 μg/mL bevacizumab (**Figure 3D**, *P* < 0.001). IgG1 (100 μg/mL), an isotype control of bevacizumab, failed to upregulate CD105 in HUVECs (**Figure 3F** and **3G**), signifying that CD105 induction was bevacizumab-specific. Regulation of CD105 by bevacizumab was consistently validated in MRMECs and bEnd.3 cells (**Figure 4C** and **4D**, *P* < 0.05).

Furthermore, protein levels of Endo-MT-related factors, including Slug (**Supplementary Figure S2A** and **S2B**), Twist (**Supplementary Figure S2A** and **S2C**), α-SMA (**Supplementary Figure S2A** and **S2D**), and N-cadherin (**Supplementary Figure S2A** and **S2E**), and inflammatory factors, including IL1B (**Supplementary Figure S2A** and **S2F**) and CCL20 (**Supplementary Figure S2A** and **S2G**), were significantly increased (*P* < 0.05) in the presence of 100 μg/mL bevacizumab.

### TGFβ1 is upregulated in HUVECs treated with 100 μg/mL bevacizumab

**Figure 5:**
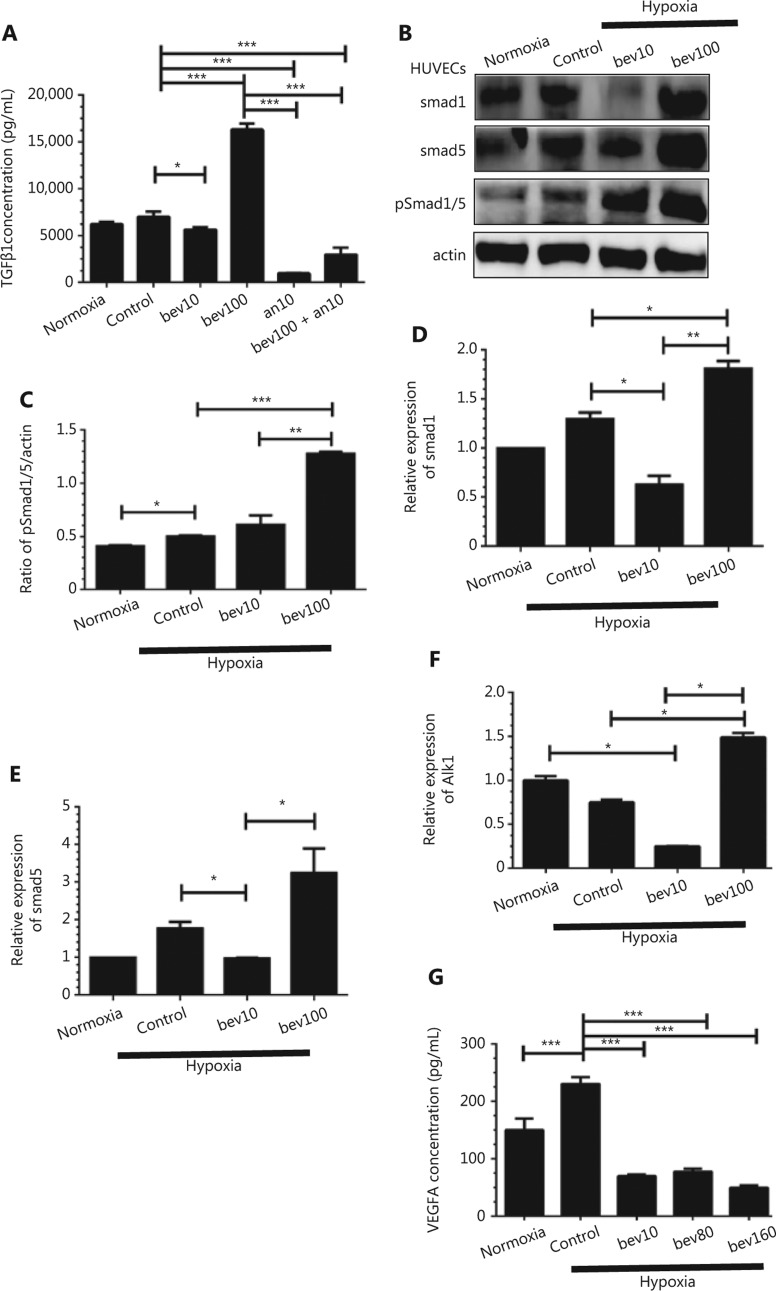
The TGFβ pathway is activated by high concentration of bevacizumab (100 μg/mL) under hypoxia conditions. (A) Concentrations of secreted TGFβ1 in supernatant determined using ELISA under hypoxia. HUVECs were treated with 10 and 100 μg/mL bevacizumab (24 h), anlotinib 10 μM (24 h), bevacizumab (100 μg/mL for 8 h) and anlotinib (10 μM for 16 h) sequentially; normoxia: normal oxygen vehicle. *P < 0.05; ***P < 0.001, one-way ANOVA. (B) Western blot showing changes in Smad1, Smad5, and pSmad1/5 protein levels (downstream factor of TGFβ and CD105) following bevacizumab treatment under hypoxia. (C) Quantitative analysis of pSmad1/5 protein levels following bevacizumab treatment. Data represent mean ± SD, *P < 0.05, **P < 0.01, ***P < 0.001; one-way ANOVA. (D–F) Quantitative analysis of Smad1, Smad5, and alk1 mRNA levels following treatment with bevacizumab under hypoxia, *P < 0.05, **P < 0.01, ***P < 0.001; one-way ANOVA. (G) Concentrations of secreted VEGFA in the supernatant under hypoxia, as determined with ELISA. HUVECs were treated with 10, 80, and 160 μg/mL bevacizumab (starvation for 12 h); normoxia: normal oxygen vehicle, *P < 0.05, **P < 0.01, ***P < 0.001; one-way ANOVA.

To examine the potential involvement of TGFβ1 in CD105 regulation, HUVECs were serum-starved for 24 h and stimulated with bevacizumab. Under hypoxia conditions, the TGFβ1 concentration was significantly increased in HUVECs treated with 100 μg/mL bevacizumab (**Figure 5A**, *P* < 0.001), along with pSmad1/5 protein levels (**Figure 5B** and **5C**, *P* < 0.001). Smad1, Smad5, and alk1 mRNA levels following treatment with bevacizumab were additionally increased under hypoxia (**Figure 5D–5F**). The observed changes in CD105, Smad1 and Smad5 protein levels were further validated in bEnd.3 cells and MRMECs (**Figure 4C** and **4D**, *P* < 0.05). Interestingly, TGFβ1 expression was also enhanced by 100 μg/mL bevacizumab under oxygen conditions (**Supplementary Figure S1C** and **S1D**, *P* < 0.0001).

### Anlotinib reverses bevacizumab-induced upregulation of CD105 in HUVECs

HUVECs, bEnd.3, and MRMECs were treated with bevacizumab (100 μg/mL) for 12 h, followed by anlotinib (10 μM), which targets multiple receptor tyrosine kinases, including VEGFR2/3, FGFR1-4, PDGFRα/β, C-Kit, and RET, for a further 12 h^24^. ECs sequentially treated with bevacizumab and anlotinib were compared with those treated with bevacizumab alone for 24 h. Our results showed a significant decrease in CD105 levels in the former group (**Figure 4A** and **4B**, *P* < 0.001), indicating that bevacizumab-induced CD105 augmentation is effectively suppressed by anlotinib. Augmentation of CD105 and Smads in MRMECs and bEnd.3 cells was similarly reversed by anlotinib (**Figure 4C** and **4D**). In HUVECs, MRMECs and bEnd.3 cells treated with anlotinib for 6 h, followed by bevacizumab for 18 h, upregulation of CD105 was also suppressed (**Supplementary Figure S3A–S3C**, *P* < 0.01), indicating that the sequence of anlotinib treatment does not influence its inhibitory effect.

**Figure 6:**
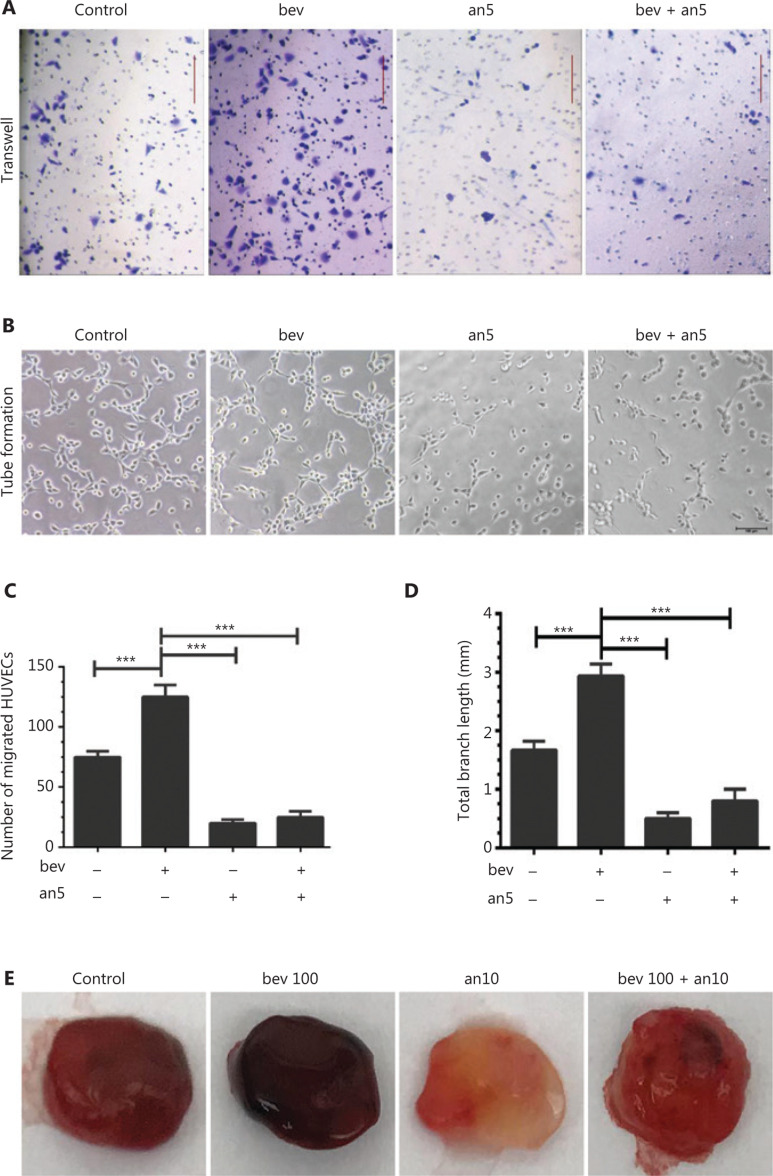
Anlotinib suppresses HUVEC migration and tube formation. (A) Typical images of HUVECs in migration assays following treatment with or without anlotinib and bevacizumab under hypoxia (an5: anlotinib 5 μM, bev: bevacizumab 100 μg/mL). Magnification, ×100. (B) Images of canal-like tubes formed by HUVECs treated with or without anlotinib and bevacizumab under hypoxia (an5: anlotinib 5 μM, bev: bevacizumab 100 μg/mL), Magnification, ×50. (C) Number of migrated HUVECs. Data represent mean ± SD, *P < 0.05, **P < 0.01, ***P < 0.001; one-way ANOVA. (D) Average total branching lengths of the capillary-like tubules formed following anlotinib and bevacizumab treatment under hypoxia. Data represent mean ± SD, *P < 0.05, **P < 0.01, ***P < 0.001; one-way ANOVA. (E) Comparison of blood vessel formation in matrigel (400 μL) plugs in female nude mice (*n* = 4 per group) between control (bevacizumab: 0 μg/mL in matrigel), bev100 (bevacizumab: 100 μg/mL in matrigel) and bev100 + an10 (100 μg/mL bevacizumab and 10 μM anlotinib in matrigel) groups. Blood and vessel structures were evident in the bev100 and control groups, but bEnd.3 cells treated with bevacizumab and anlotinib displayed no obvious vessel structures in the matrigel. Mixed matrigel containing bEnd.3 cells, bevacizumab, and VEGFA was subcutaneously injected into mice, followed by intraperitoneal injection with 0, 5, and 50 mg/kg bevacizumab twice a week for 9 days. The image shows bEnd.3 matrigel separated from mice, with darker red indicative of higher blood content in the gel.

Following treatment of HUVECs with anlotinib (5 μM) in hypoxia conditions, migration (**Figure 6A** and **6C**, *P* < 0.001) and tube formation abilities (**Figure 6B** and **6D**, *P* < 0.001) were significantly decreased. Sequential treatment of HUVECs with bevacizumab and anlotinib resulted in marked downregulation of tube formation and migration abilities, compared to cells treated with bevacizumab alone (**Figure 6B** and **6D**, *P* < 0.001). Consistent with our *in vitro* findings, anlotinib reversed the pro-angiogenic effects of bevacizumab in bEnd.3 cells *in vivo* (**Figure 6E**). The blood content was higher in more dense vessel structures in bEnd.3 cell matrigel plugs treated with 100 μg/mL bevacizumab and significantly decreased in vessel structures in matrigel plugs treated with anlotinib.

### siRNA targeting CD105 suppresses migration and proliferation of HUVECs and downregulates downstream factors

**Figure 7:**
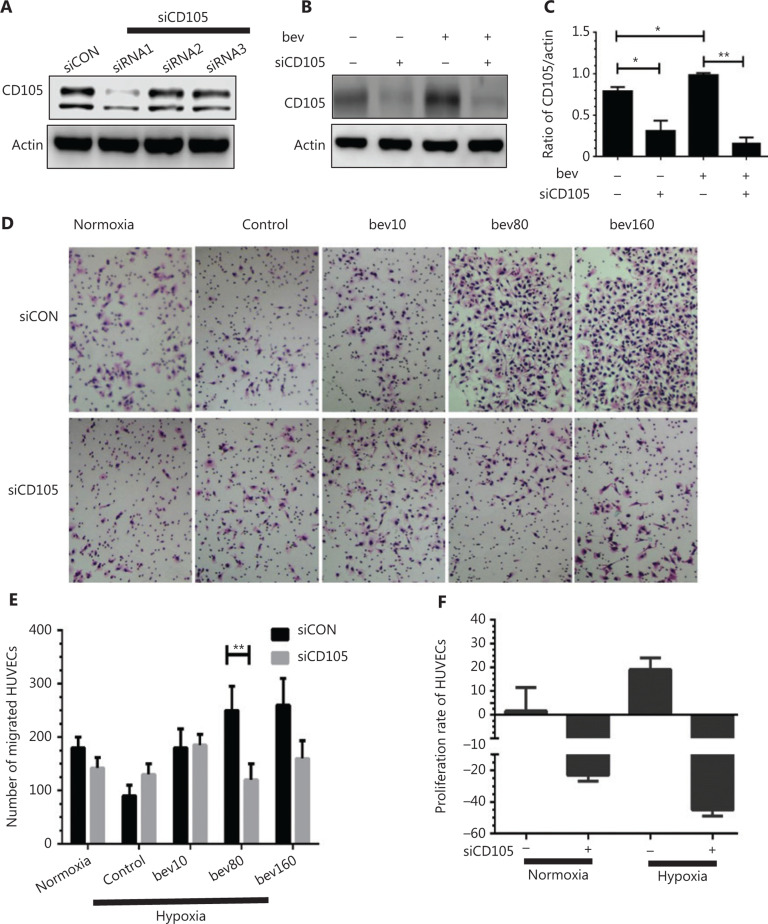
CD105 siRNA suppresses CD105 expression as well as migration and proliferation of HUVECs promoted by bevacizumab. (A) CD105 protein levels following siCD105 (CD105 siRNA) treatment determined *via* Western blot. The CD105 siRNA sequences are as follows: siRNA1, 5′-CCA UGA CCC UGG UAC UAA A-3′ and 3′-GGU ACU GGG ACC AUG AUU U-5′; siRNA2, 5′-UGA CCU GUC UGG UUG CAC ATT-3′ and 5′-UGU GCA ACC AGA CAG GUC AGG-3′; siRNA3, 5′-GAG GUG ACA UAU ACC ACU A-3′, 5′-CUC CAC UGU AUA UGG UGA U-3′; and siCON, 5′-UUC UCC GAA CGU GUC ACG UTT-3′ and 5′-ACG UGA CAC GUU CGGAGAATT-3′. CD105 was markedly downregulated by siRNA1, which was selected for subsequent experiments. (B) Western blot assessment of CD105 protein levels following bevacizumab treatment in the presence or absence of siCD105 (CD105 siRNA). (C) Densitometry analysis of CD105 protein levels from B. Data represent mean ± SD, *P < 0.05, **P < 0.01; Student’s *t*-test. (D) Typical images of migrated HUVECs in transwell assays following bevacizumab treatment in the presence or absence of CD105 siRNA (siCD105); normoxia: normal oxygen vehicle, control: hypoxia, bevacizumab 0 μg/mL, bev10, bev80, bev160: bevacizumab 10 μg/mL, 80 μg/mL, 160 μg/mL. (E) Number of migrated HUVECs. Data represent mean ± SD, **P < 0.01; Student’s *t-*test. (F) Proliferation rate of HUVECs decreased following treatment with siRNAs targeting CD105 under both normal and hypoxia conditions. Data represent mean ± SD.

CD105 was depleted using siRNA, even with bevacizumab stimulation, as evident from western blot analysis (**Figure 7A–7C**). Downstream factors of CD105 and inflammatory factors, such as CCL20 and IL1B, were additionally decreased (**Supplementary Figure S4A–S4C**, *P* < 0.01), along with Endo-MT-related factors, such as Twist, N-cadherin, and Snail (**Supplementary Figure S4D–S4F**, *P* < 0.01). Migration of HUVECs treated with 80 μg/mL bevacizumab and positive siRNA under hypoxia conditions was markedly decreased, compared with that in the negative siRNA group (**Figure 7D** and **7E**, *P* < 0.01). Proliferation of HUVECs was additionally suppressed in both normal and hypoxia conditions (**Figure 7F**), clearly supporting stimulatory effects of CD105 on both cell migration and proliferation.

## Discussion

Bevacizumab has been developed as a key anti-angiogenic agent to reinforce the efficacy of chemotherapy with recognized benefits in the clinic^25,26^. However, a number of studies have reported negative results without significant prolongation of OS^5^. These findings highlight a common problem of single-target anti-angiogenic drugs, i.e., triggering of hypoxia and subsequent activation of tumor cells, initiating interstitial-epithelial transformation and vascular mimicry^27,28^, leading to therapeutic failure. Bevacizumab neither decreased tumor growth nor improved survival of mice bearing orthotopic or endogenous glioma while exacerbating brain tumor invasion^7,8,10,29^. Some tumors develop resistance, even after adequate anti-angiogenic therapy^9,10,27^. In many reports^30^, resistance is ascribed to augmentation of HIF, which promotes tumor cell invasion. However, few investigations to date have focused on the potential role of vascular endothelial cells (ECs) in resistance. Tumors can “hijack” and remodel normal vessels, convert normal ECs to “aberrant” cells^31^, and mobilize precursor ECs in the circulation to form vasculatures^32^. Accordingly, enhanced circulating CD105+-activated ECs are indicative of resistance to anti-vascular drugs and tumor development^33^. These findings strongly suggest that ECs can be activated, which contribute to resistance to anti-angiogenic therapy. Results from current study demonstrate that bevacizumab promotes tube formation and migration by HUVECs in hypoxia, and activates the ECs through VEGF-independent pathways.

The optimal concentration of bevacizumab determined from the cell experiments was approximately similar to the clinical dose. In the clinic, the plasma concentration of bevacizumab is reported to reach 136.3 μg/mL after treatment with 7.5–15 mg/kg bevacizumab^23^. The conversion is calculated as follows: at a standard patient weight and blood volume of 60 kg and 4 L, respectively, plasma concentration was 60*(7.5 to 15)/4 mg/L = 112.5–225.0 mg/L (i.e., 112.5–225.0 μg/mL). However, increased invasion and metastases for some malignancies have been documented upon administration of the standard dose^34^, suggesting that the standard dose may not benefit every individual and the dose for the whole body may not be always proper rather than excessive concentration in all tumor lesions. We believe that the “relatively excessive concentration” in local lesions due to heterogeneity of tumors activates ECs. However, reduction of the therapeutic dose is not a feasible option. The main purpose of this study was to explore whether “improper treatment” could activate HUVECs, determine the underlying pathways, and identify potential markers on vascular endothelial cells that could be effectively utilized to indicate anti-angiogenic drug resistance.

VEGF-A signaling is the established canonical pathway of angiogenesis. However, other alternative mechanisms exist^35,36^, including PDGF, TGFβ, and FGF pathways. Blockage of VEGF-A in glioblastoma has been shown to increase MET activity in a hypoxia-independent manner, in turn, enhancing tumor invasion^37^. In a murine glioma model, TGFβ activation mediated escape from VEGF inhibition^38^. Experiments from the current study mainly focused on whether HUVECs can be stimulated independently of VEGF *via* the TGFβ-CD105-Smad pathway.

Migration of HUVECs treated with high concentrations of bevacizumab in normoxia conditions was not increased (**Supplementary Figure S1A** and **S1B**). In HUVECs subjected to hypoxia only, migration was not significantly increased (**Figure 1A** and **1C**) either, clearly indicating that either hypoxia or bevacizumab alone is not sufficient to activate HUVECs, while hypoxia and bevacizumab acted synergistically to promote migration of HUVECs through upregulation of TGFβ1-CD105 (**Figures 3A** and **5A**). Canonically, tumor cell invasion is often attributed to stimulation of hypoxia^39,40^. However, in our experiments, when HUVECs were treated with a high concentration of bevacizumab in normoxia conditions, TGFβ1 was also elevated (**Supplementary Figure S1C** and **S1D**), consistent with results obtained under hypoxia (**Figure 5A**) and our recent report^41^. Hypoxia alone failed to increase TGFβ1 (**Figure 5A**), suggesting that signaling pathways other than HIF-α activate ECs. However, we hypothesize that activation of HUVECs is a consequence of synergistic effects based on earlier evidence of elevated TGFβ by hypoxia. TGFβ activation was additionally confirmed in MRMECs and bEnd.3 cells.

CD105, an angiogenesis marker, was elevated by high concentrations of bevacizumab, both *in vivo* and *in vitro*, which was validated in MRMECs and bEnd.3 cells (**Figure 4C** and **4D**). We further confirmed that this effect was specifically caused by bevacizumab but not control protein IgG1 (**Figure 3F**). Knockdown of CD105 was closely associated with downregulation of downstream inflammatory (CCL20 and IL1B) and Endo-MT-related factors (Snail, N-cadherin, Twist) (**Supplementary Figure S4**), which are responsible for cell migration, adhesion, and vessel formation^42–44^. Transfection with siCD105 suppressed HUVEC migration (**Figure 7D–7E**), suggesting that CD105 is a key contributory factor in endothelial cell activation. Since CD105 plays significant roles in angiogenesis, inflammation, and cancer development^45,46^, our results may partially explain the mechanism underlying vasculature endothelial cell resistance to anti-angiogenesis agents. ECs with properties of mesenchymal cells (**Supplementary Figure S2A**), termed Endo-MT^12^, play important roles in neointima formation. Endo-MT-derived cells promote tumor development by secreting specific proteins^47^. Moreover, ECs migrate to tumor sites and form vasculatures that favorably promote tumor growth^48,49^. In the present study, in HUVECs treated with high concentrations of bevacizumab, the vessel-like structure became dispersed and discontinuous, termed “co-opted vasculature”. This co-opted vasculature has been shown to exacerbate hypoxia to stimulate tumor cells to further release VEGF and enhance resistance to anti-angiogenesis agents^14,50^.

In our experiments, VEGF-A was completely blocked using bevacizumab (**Figure 5G**), signifying that activation of HUVECs is not VEGF-A-dependent. To our knowledge, this is the first study to demonstrate that HUVECs can be activated through TGFβ1-CD105-Smad signaling triggered by bevacizumab independently of the VEGF pathway. These results highlight the common shortcomings of single-target drugs, i.e., when one signaling pathway of VEGF was shut down the alternative byways may be stimulated. TGFβ1 can activate downstream CD105^51^ and the classical Smad cascade^52^. Concordantly, elevation of activated circulating endothelial cells (aCEC) positive for CD105 is an indicator of NSCLC resistance to anti-angiogenesis and poor prognosis^53^. Our findings are consistent with a previous report that CD105 is upregulated in hypoxia conditions *via* activation of the MAPK pathway (including p38 and JNK)^54^.

Upregulation of CD105 by bevacizumab was reversed by anlotinib, in keeping with recent results obtained by our group^41^. These results indicate that multi-target drugs can attenuate resistance through suppressing multiple byway signaling pathways initiated by a single-target inhibitor^24,55–57^. Although the mechanisms by which bevacizumab and anlotinib exert their activities on TGFβ remain to be established, previous studies suggest that FGF signaling is activated when VEGF signaling is blocked^58^ and that under conditions of bevacizumab-induced decrease in VEGF, angiogenin and bFGF levels are significantly increased^59^. Meanwhile, FGF2 cooperates with TGFβ to promote motility and proliferation of endothelial cells^60^, which may explain the observed bevacizumab-mediated activation. Blockage of FGF signaling by anlotinib^56^ could underlie inactivation of HUVECs. Furthermore, anlotinib inhibited tube formation and migration of HUVECs in our experiments.

Our study has a number of limitations that should be taken into consideration. Firstly, the precise mechanisms by which bevacizumab and anlotinib affect TGFβ1 signaling in cells remain unclear. Moreover, expression changes in CD105, TGFβ1, VEGF, HIF-1a and tumor vasculature in the presence of low and high doses of bevacizumab in nude mouse models and clinical specimens require further elucidation. Our future studies will investigate whether bevacizumab interacts directly with TGFβ1.

## Conclusions

In conclusion, higher concentrations of bevacizumab (80–160 μg/mL) can activate the TGFβ1-CD105-Smad pathway, promoting migration and tube formation of HUVECs under hypoxia (**Supplementary Figure S5**). CD105 may serve as a potential marker of resistance to anti-angiogenesis drugs. Anlotinib effectively reverses the effects of bevacizumab.
