# Supplementary material for: Bevacizumab promotes active biological behaviors of human umbilical vein endothelial cells by activating TGFβ1 pathways via off-VEGF signaling
Source: Cancer Biol Med. 2020 May 15;17(2):418–32. doi: 10.20892/j.issn.2095-3941.2019.0215 (PMC7309466; doi:10.20892/j.issn.2095-3941.2019.0215)
Supplement: Supplementary file 1 [file cbm-17-418-s001.pdf]

Supplementary materials

Table S1 Primers used in this study

Gene	Primer sequence
Smad1	
Forward	5′-ACAGTCTGTGAACCATGGATTTGA-3′
Reverse	5′-TGAGGTGAACCCATTTGAGTAAGAA-3′
Smad5	
Forward	5′-GCTTTCATCCCACCACTGTCTGTA-3′
Reverse	5′-CCTGCCGGTGATATTCTGCTC-3′
CD105	
Forward	5′-ACCACTTCGGAAAAAGG-3′
Reverse	5′-GCTGAAACGTGGGTCG-3′
alk1	
Forward	5′-GTCAAGAAGCCTCCAGAAC-3′
Reverse	5′-CATCAACTCAGGCTTCGGG-3′
GAPDH	
Forward	5′-CCTTCATTGACCTCAACTA-3′
Reverse	5′-GGAAGGCCATGCCAGTGAGC-3′
siCD105-1	
Forward	5′-CCA UGA CCC UGG UAC UAA A-3′
Reverse	5′-GGU ACU GGG ACC AUG AUU U-3′
siCD105-2	
Forward	5′-UGACCUGUCUGGUUGCACATT-3′
Reverse	5′-UGUGCAACCAGACAGGUCAGG-3′
siCD105-3	
Forward	5′-GAG GUG ACA UAU ACC ACU A-3′
Reverse	5′-CUC CAC UGU AUA UGG UGA U-3′
Negative control	
Forward	5′-UUCUCCGAACGUGUCACGUTT-3′
Reverse	5′-ACGUGACACGUUCGGAGAATT-3′

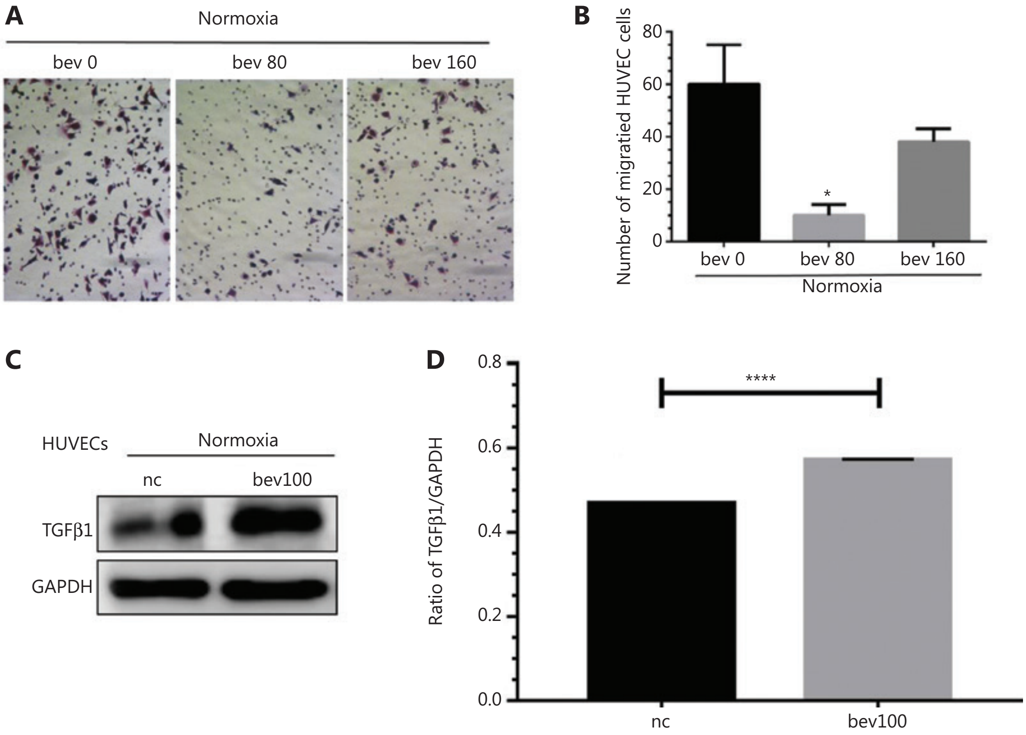

Figure S1 High concentration of bevacizumab (100 μg/mL) fails to promote migration of HUVECs in normoxia conditions but enhances TGFβ1. (A) Typical images of migrated HUVECs pretreated with bevacizumab under normoxia; bev0, bev80, bev160 (bevacizumab 0 μg/mL, 80 μg/mL, 160 μg/mL); normoxia: 21% O_2, 5% CO_2, 74% N_2, magnification, ×100. (B) Quantitative analysis of migrated HUVECs treated with different doses of bevacizumab in normoxia conditions. Data represent mean ± SD of three independent experiments; *$P < 0.05$; one-way ANOVA. Without bar: compared with other groups. (C) Expression of TGFβ1 in HUVECs under high-dose bevacizumab treatment in normoxia. (D) Expression of TGFβ1 in MRMECs under high-dose bevacizumab treatment in normoxia, which is consistent with data obtained using HUVECs. ****$P < 0.0001$.

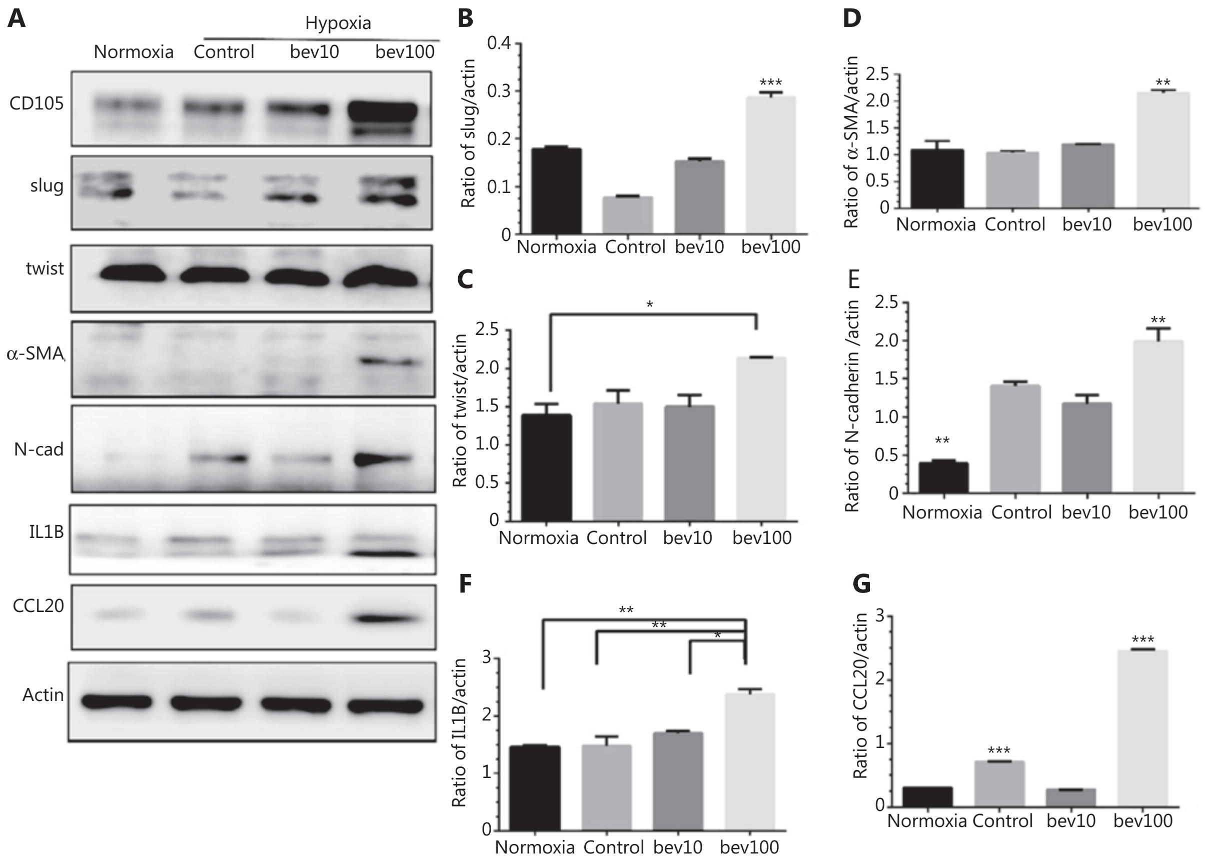

Figure S2 High concentration of bevacizumab (100 μg/mL) enhances expression of downstream factors of CD105 in HUVECs. (A) Western blot showing changes in protein levels of CD105 and downstream factors following bevacizumab treatment under hypoxia (control: bevacizumab 0 μg/mL, bev10: bevacizumab 10 μg/mL, bev100: bevacizumab 100 μg/mL; normoxia: normal oxygen vehicle). (B–E) Quantitative analysis of Slug, Twist, αiSMA, and N-cadherin (Endo-MT factor) protein levels following bevacizumab treatment. Data represent mean ± SD, **P* < 0.05, ***P* < 0.01, ****P* < 0.001; one-way ANOVA. Without bar: compared with other groups. (F, G) Quantitative analysis of protein levels of IL1B and CCL20 (inflammatory factors) following bevacizumab treatment. Data represent mean ± SD, **P* < 0.05, ***P* < 0.01, ****P* < 0.001; one-way ANOVA. Without bar: compared with other groups.

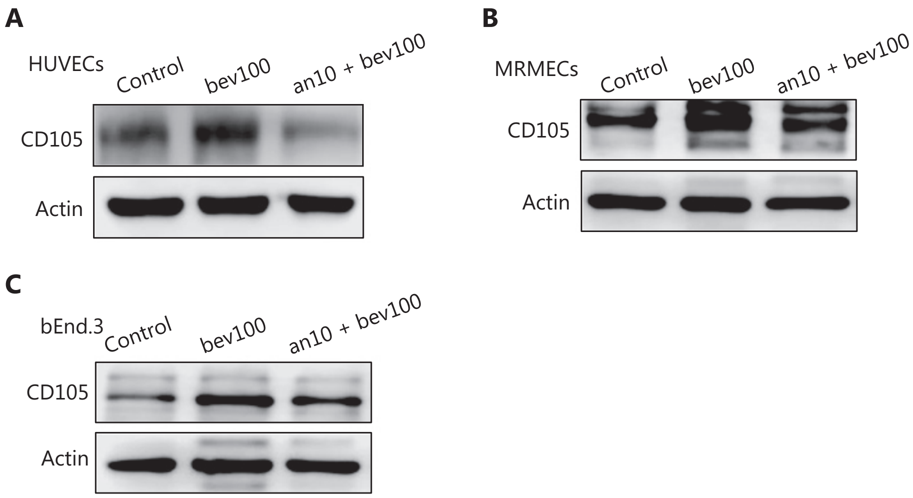

Figure S3 Anlotinib reverses upregulation of CD105 induced by bevacizumab. (A-C) HUVECs, bEnd.3 cells, and MRMECs were treated with bevacizumab (100 μg/mL, 24 h) and bevacizumab (100 μg/mL, 18 h) after pretreatment with anlotinib (10 μM, 6 h) under hypoxia conditions.

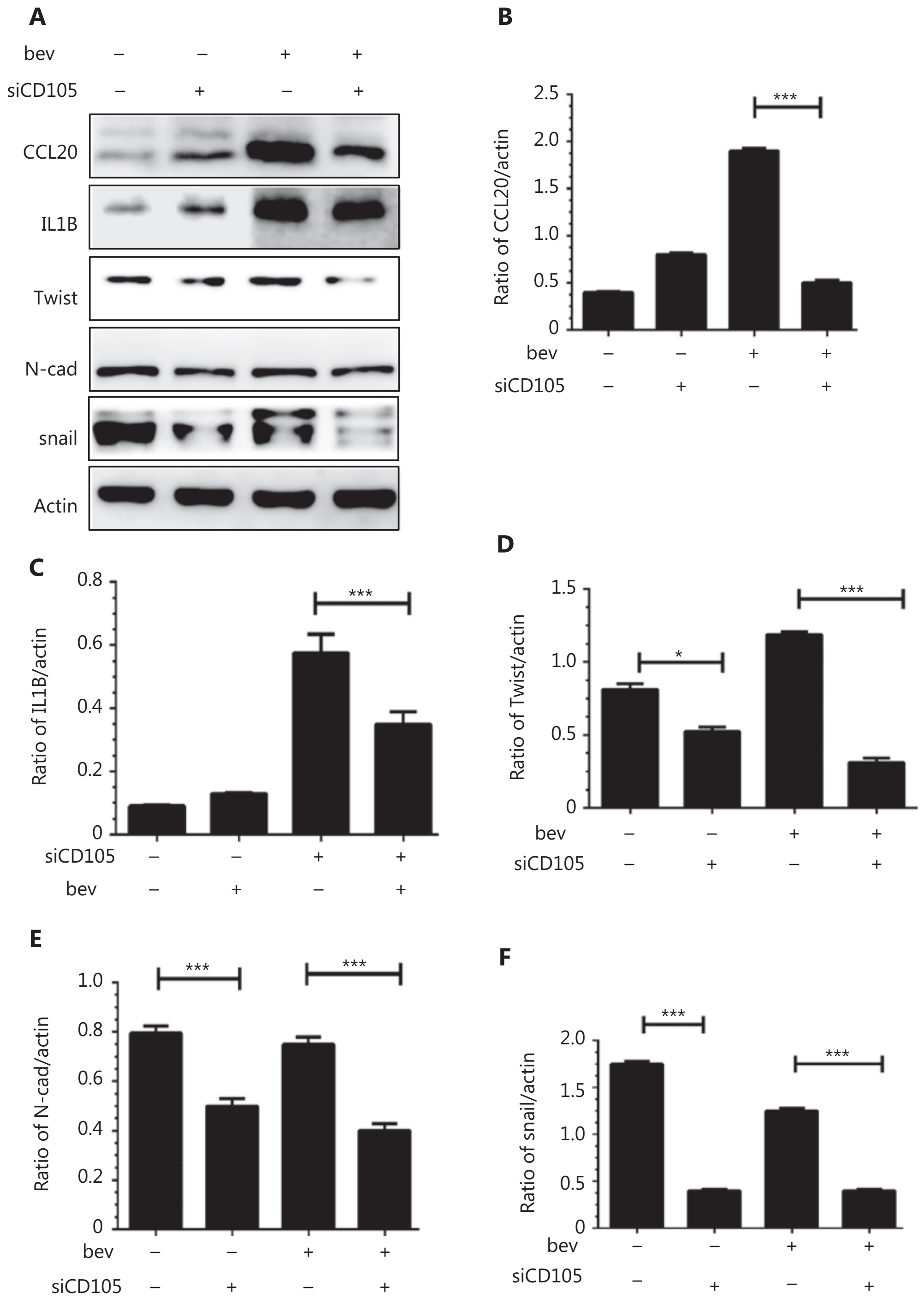

Figure S4 CD105 siRNA attenuates expression of downstream factors of CD105 in HUVECs. (A) Western blot analysis of protein levels of endo-MT factors (N-cadherin, Snail, Twist) and inflammatory factors (CCL20, IL1B) following bevacizumab treatment in the presence or absence of siCD105 (CD105 siRNA). (B–F) Densitometry analysis of CCL20, IL1B, Twist, N-cad, and Snail protein levels shown in (A). Data represent mean ± SD, *$P < 0.05$, **$P < 0.01$, ***$P < 0.001$; Student's *t*-test.

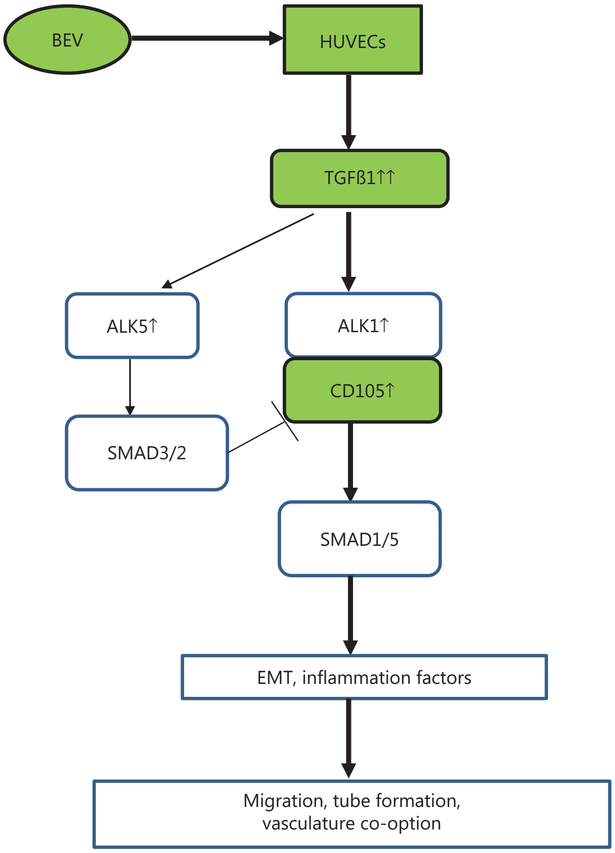

Figure S5 Potential molecular basis of activation of HUVECs triggered by bevacizumab. Bevacizumab activates HUVECs through the TGFβ1-CD105-Smad pathway, posing a potential risk of drug resistance.
